# Solitary Cavitary Right Upper Lobe Lesion Revealing Disseminated Cryptococcosis in a Kidney Transplant Recipient: A Diagnostic Challenge

**DOI:** 10.1155/crpu/5586947

**Published:** 2026-05-20

**Authors:** Dawit Worku, Michael Convertino, Alex Saunders, Alehegn Gelaye, Ahmad Rachid

**Affiliations:** ^1^ Division of Pulmonary and Critical Care Medicine, Henry Ford Health Providence Hospital, Michigan State University College of Human Medicine, Southfield, Michigan, USA, msu.edu

**Keywords:** cavitary lung lesion, cryptococcal antigen, cryptococcosis, kidney transplant, opportunistic fungal infection, pulmonary cryptococcosis

## Abstract

Pulmonary cryptococcosis is an opportunistic fungal infection that primarily affects immunocompromised individuals, including solid organ transplant recipients. Its radiologic manifestations are diverse and may closely mimic malignancy or other chronic pulmonary infections, often leading to diagnostic uncertainty. We report a case of disseminated cryptococcosis presenting as a solitary cavitary pulmonary lesion in a kidney transplant recipient receiving chronic immunosuppressive therapy. Imaging findings initially raised concern for malignancy. Cytologic evaluation demonstrated fungal elements favoring *Histoplasma*, complicating the diagnostic process. However, microbiologic cultures and cryptococcal antigen testing confirmed *Cryptococcus neoformans*. Cerebrospinal fluid analysis demonstrated central nervous system involvement. The patient was treated with induction antifungal therapy followed by consolidation and maintenance therapy, with clinical and radiographic improvement. This case highlights the diagnostic challenges of pulmonary cryptococcosis and underscores the importance of integrating imaging, pathology, microbiology, and antigen testing to establish the diagnosis in immunocompromised patients.

Summary

• Cavitary pulmonary cryptococcosis may mimic malignancy.
• Cytologic interpretation may be misleading in fungal infections.
• Culture and antigen testing are essential for definitive diagnosis.
• CNS involvement should be evaluated early.
• Multimodal diagnostic integration improves clinical accuracy.

## 1. Introduction

Cryptococcosis is an opportunistic fungal infection caused primarily by *Cryptococcus neoformans* and *Cryptococcus gattii*. Infection typically follows inhalation of environmental fungal spores, with the lungs serving as the initial site of involvement. In immunocompromised individuals, particularly solid organ transplant recipients, hematogenous dissemination may occur, most commonly involving the central nervous system and resulting in cryptococcal meningitis [1, 2].

Pulmonary cryptococcosis demonstrates a broad spectrum of radiologic manifestations, including nodules, mass‐like lesions, consolidation, and, less commonly, cavitary lesions [3–5]. These findings frequently overlap with malignancy, tuberculosis, and other fungal infections, making diagnosis particularly challenging in immunocompromised hosts.

Although pulmonary cryptococcosis is well recognized in transplant populations, certain presentations remain diagnostically complex. This case is notable for a solitary cavitary lesion initially concerning for malignancy, cytologic findings suggesting an alternative fungal etiology, and subsequent confirmation of disseminated cryptococcosis through combined microbiologic and antigen‐based testing. It illustrates the importance of a structured and integrated diagnostic approach.

## 2. Case Presentation

A woman in her 60s with a history of end‐stage renal disease status post–kidney transplantation presented with progressive fatigue, dizziness, and gait instability over several days.

She had undergone three kidney transplants (1999, 2008, and 2016). Her allograft function remained stable, with a baseline creatinine of approximately 1.5–1.6 mg/dL.

Her maintenance immunosuppressive regimen included tacrolimus 5 mg twice daily, mycophenolate mofetil 500 mg twice daily, and prednisone 10 mg daily. She denied recent medication changes, rejection episodes, or corticosteroid escalation.

**Figure 1:**
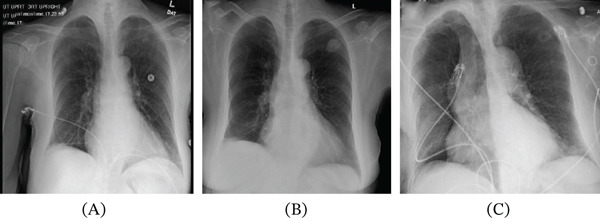
Chest radiographs demonstrating the evolution of the pulmonary lesion. (A) Chest radiograph obtained approximately 2 years prior, demonstrating a subtle right upper lobe opacity. (B) Chest radiograph obtained at presentation demonstrating a more prominent right upper lobe lesion. (C) Follow‐up chest radiograph at approximately 6 weeks demonstrating an interval decrease in lesion size and density, consistent with a resolving infectious process.

**Figure 2:**
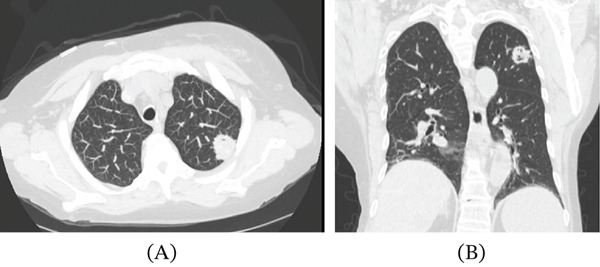
CT imaging of the chest demonstrating cavitary morphology of the lesion. (A) Axial CT image showing a cavitary lesion within the right upper lobe. (B) Coronal CT reconstruction demonstrating the cavitary nature of the lesion.

Initial chest radiography demonstrated a right upper lobe opacity (Figure 1A–C). Computed tomography of the chest revealed a cavitary lesion measuring approximately 2.5 × 2.4 cm with irregular margins and central cavitation (Figure 2A,B). These findings raised concern for malignancy, mycobacterial infection, or invasive fungal disease.

Review of prior imaging demonstrated a subtle abnormality in the same region (Figure 1A). While a definitive temporal relationship cannot be established, this raised the possibility of a chronic or indolent process.

**Figure 3:**
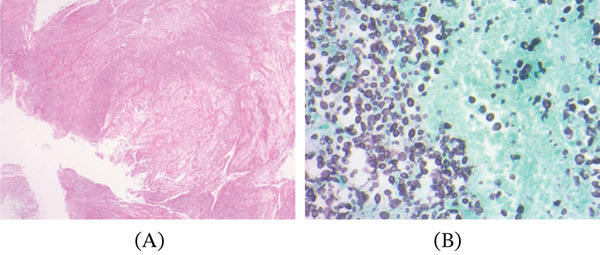
Histopathologic findings of pulmonary cryptococcosis. (A) Low‐power microscopic examination demonstrating necrotic debris and inflammatory infiltrates within lung tissue. (B) Gomori methenamine silver stain highlighting yeast forms consistent with cryptococcal organisms.

Bronchoscopy with bronchoalveolar lavage and fine‐needle aspiration was performed early during hospitalization. Cytologic evaluation demonstrated fungal elements with small oval yeast forms and narrow‐based budding (Figure 3A,B), initially interpreted as favoring *Histoplasma* species; however, definitive speciation was not possible based on morphology alone.

Microbiologic cultures from bronchoalveolar lavage subsequently grew *Cryptococcus neoformans* and rare *Aspergillus fumigatus*. Serum cryptococcal antigen testing was strongly positive with a titer of 1:2560. Serum *Aspergillus* antigen and serum beta‐D‐glucan testing were performed and were negative (Table 1).

**Table 1:** Key laboratory and diagnostic findings.

Parameter	Result	Reference range
Serum cryptococcal antigen	Positive	Negative
Serum cryptococcal antigen titer	1:2560	Negative
CSF white blood cell count	161 cells/*μ*L	< 5 cells/*μ*L
CSF cryptococcal antigen	Positive	Negative
CSF cryptococcal antigen titer	1:640	Negative
CSF protein	355.0 mg/dL	15–45 mg/dL
CSF glucose	64 mg/dL	45–80 mg/dL
BAL culture	*Cryptococcus neoformans*	Negative
CSF culture	*Cryptococcus neoformans*	Negative
*Aspergillus* antigen	Negative	Negative
Beta‐D‐glucan	Negative	Negative

Given the patient′s neurologic symptoms, a lumbar puncture was performed shortly after admission. Opening pressure at the time of the initial lumbar puncture was 18 cm H_2_O, with a closing pressure of 10 cm H_2_O. Cerebrospinal fluid analysis demonstrated a white blood cell count of 161 cells/*μ*L, elevated protein, and decreased glucose. Cryptococcal antigen was positive with a titer of 1:640, and CSF cultures grew *Cryptococcus neoformans*, confirming disseminated cryptococcosis with central nervous system involvement.

A repeat lumbar puncture performed during hospitalization demonstrated an opening pressure of 11 cm H_2_O and a closing pressure of 8 cm H_2_O, along with a decrease in CSF inflammatory markers and negative follow‐up cultures, consistent with treatment response.

Magnetic resonance imaging of the brain with and without contrast was performed in the setting of neurological symptoms. Imaging demonstrated no pathologic enhancement, mass lesion, or evidence of acute or subacute ischemia. Moderate chronic periventricular and deep white matter changes were noted without findings suggestive of focal cryptococcal involvement.

The patient was treated with induction antifungal therapy consisting of liposomal Amphotericin B (250 mg daily) and flucytosine (2000 mg every 12 h) for approximately 3 weeks. Laboratory monitoring included renal function, complete blood counts, and liver function tests. Therapy was well tolerated without major adverse effects.

Following induction therapy, she was transitioned to fluconazole 400 mg daily (dose adjusted for renal function) for consolidation therapy over 8 weeks, followed by maintenance therapy with fluconazole 200 mg daily for 1 year.

The patient demonstrated clinical improvement and was discharged home with close outpatient follow‐up. Follow‐up chest imaging several weeks after initiation of therapy demonstrated interval reduction in lesion size to approximately 1.7 × 1.1 cm with decreased density and persistent cavitation, consistent with a resolving infectious process (Figure 1C).

## 3. Discussion

Cryptococcosis is a clinically significant opportunistic infection in solid organ transplant recipients due to impaired cell‐mediated immunity associated with chronic immunosuppressive therapy. Although the reported incidence is relatively low, typically ranging from 2% to 3%, the consequences can be severe, particularly when dissemination occurs [6, 7]. Notably, infection may develop even in patients with stable graft function and no recent escalation of immunosuppression, as demonstrated in this case.

Pulmonary involvement is often the initial manifestation of cryptococcal infection, as inhalation represents the primary route of exposure. However, the clinical presentation is highly variable. Radiologic findings are frequently nonspecific and may include nodules, consolidation, or mass‐like lesions, and less commonly cavitary lesions [3–5]. Cavitary disease is particularly important because it broadens the differential diagnosis to include malignancy, mycobacterial infection, and other invasive fungal processes [8]. In this case, the solitary cavitary lesion raised strong concern for malignancy, highlighting a common diagnostic challenge in immunocompromised patients.

The interpretation of prior imaging further complicates clinical decision‐making. Although a subtle abnormality was present in the same region on earlier imaging, a definitive temporal relationship cannot be established. However, the interval decrease in lesion size following antifungal therapy supports an infectious etiology rather than malignancy. This underscores the importance of longitudinal imaging in evaluating indeterminate pulmonary lesions.

A key diagnostic challenge in this case was the discrepancy between cytologic findings and the final diagnosis. *Histoplasma* typically appears as small intracellular yeast forms within macrophages, whereas *Cryptococcus* is generally larger, encapsulated, and often extracellular. However, in limited cytologic specimens, particularly when capsule visualization is suboptimal, morphologic overlap may lead to provisional misclassification. This limitation underscores that cytology alone is insufficient for definitive diagnosis and should be interpreted in conjunction with culture and antigen‐based testing [3]. Definitive diagnosis in this case was established through microbiologic cultures and antigen testing. Both bronchoalveolar lavage and cerebrospinal fluid cultures grew *Cryptococcus neoformans*, while serum and CSF cryptococcal antigen testing were strongly positive. Antigen testing is highly sensitive and specific and plays a central role in diagnosis, particularly in disseminated disease [1, 9].

The detection of rare *Aspergillus fumigatus* in respiratory cultures illustrates an additional diagnostic consideration. In immunocompromised patients, colonization or incidental detection of organisms may occur. The absence of supporting serologic evidence, including negative *Aspergillus* antigen and beta‐D‐glucan testing, suggested that *Aspergillus* was not the primary pathogen. This emphasizes the need to interpret microbiologic findings within the broader clinical context.

Central nervous system involvement is the most severe manifestation of cryptococcosis and requires prompt recognition. In this case, early lumbar puncture confirmed cryptococcal meningitis, with elevated CSF white blood cell count, abnormal protein and glucose levels, and positive antigen testing. Repeat cerebrospinal fluid analysis demonstrated improvement in inflammatory parameters and clearance of cultures, consistent with therapeutic response. Serial CSF evaluation is an important component of management, as it helps assess treatment efficacy and guide the duration of therapy [1, 9].

Management of disseminated cryptococcosis with CNS involvement follows a staged antifungal approach. Current guidelines recommend induction therapy with Amphotericin B in combination with flucytosine, followed by consolidation and maintenance therapy with fluconazole [1, 10]. In this case, treatment was administered with close monitoring, including renal function, hematologic parameters, and liver function tests. The patient tolerated therapy without major adverse effects, which is notable given the potential toxicities associated with these medications.

Follow‐up imaging further supports the diagnosis and effectiveness of therapy. Reduction in lesion size and density over time is consistent with resolution of infection and helps differentiate infectious processes from malignancy. This highlights the importance of imaging not only in diagnosis but also in monitoring response to treatment.

Radiologic–pathologic correlation provides additional insight into disease pathophysiology. Cavitary lesions in cryptococcosis likely result from fungal proliferation within the lung parenchyma, leading to inflammatory response, granuloma formation, and tissue necrosis. Recognition of this correlation is important when evaluating cavitary lung lesions in immunocompromised patients [8].

Although pulmonary cryptococcosis is a recognized entity in transplant populations, this case illustrates several important clinical and diagnostic lessons. First, pulmonary cryptococcosis may present as a solitary cavitary lesion closely mimicking malignancy. Second, cytologic interpretation alone may be insufficient for definitive diagnosis due to overlapping fungal morphology. Third, a combined diagnostic strategy incorporating imaging, microbiology, and antigen testing is essential. Fourth, detection of multiple organisms requires careful clinical interpretation. Finally, early recognition and appropriate antifungal therapy can lead to favorable outcomes, even in cases of disseminated disease.

## 4. Conclusion

Pulmonary cryptococcosis may present as a cavitary lung lesion mimicking malignancy, particularly in immunocompromised patients. This case underscores the importance of integrating imaging, cytology, microbiology, and antigen testing to establish an accurate diagnosis. Early recognition and appropriate antifungal therapy can lead to favorable clinical and radiographic outcomes.

## Funding

No funding was received for this manuscript.

## Ethics Statement

Informed consent was obtained from the patient for participation in this case report. In accordance with institutional policies, formal ethics committee approval was not required for this type of publication.

## Consent

The patient provided written consent for the publication of this case report and the associated clinical images.

## Conflicts of Interest

The authors declare no conflicts of interest.

## Data Availability

The data that support the findings of this study are available from the corresponding author upon reasonable request.
